# Author Correction: A proteomic landscape of diffuse-type gastric cancer

**DOI:** 10.1038/s41467-018-04166-z

**Published:** 2018-05-08

**Authors:** Sai Ge, Xia Xia, Chen Ding, Bei Zhen, Quan Zhou, Jinwen Feng, Jiajia Yuan, Rui Chen, Yumei Li, Zhongqi Ge, Jiafu Ji, Lianhai Zhang, Jiayuan Wang, Zhongwu Li, Yumei Lai, Ying Hu, Yanyan Li, Yilin Li, Jing Gao, Lin Chen, Jianming Xu, Chunchao Zhang, Sung Yun Jung, Jong Min Choi, Antrix Jain, Mingwei Liu, Lei Song, Wanlin Liu, Gaigai Guo, Tongqing Gong, Yin Huang, Yang Qiu, Wenwen Huang, Tieliu Shi, Weimin Zhu, Yi Wang, Fuchu He, Lin Shen, Jun Qin

**Affiliations:** 1 The Joint Laboratory of Translational Medicine, National Center for Protein Sciences (Beijing) and Peking University Cancer Hospital, State Key Laboratory of Proteomics, Institute of Lifeomics, 102206 Beijing, China; 2 Key Laboratory of Carcinogenesis and Translational Research (Ministry of Education), Peking University Cancer Hospital & Institute, 100142 Beijing, China; 3 State Key Laboratory of Genetic Engineering, Human Phenome Institute, Institutes of Biomedical Sciences, and School of Life Sciences, Zhongshan Hospital, Fudan University, 200433 Shanghai, China; 4 Center for Bioinformatics, East China Normal University, 200241 Shanghai, China; 5 Human Genome Sequencing Center, Department of Molecular and Human Genetics, Baylor College of Medicine, Houston, TX 77030 USA; 6 General Hospital of Chinese People’s Liberation Army, 100853 Beijing, China; 7 Affiliated Hospital of Academy of Military Medical Sciences, 100071 Beijing, China; 8 Alkek Center for Molecular Discovery, Verna and Marrs McLean Department of Biochemistry and Molecular Biology, Department of Molecular and Cellular Biology, Baylor College of Medicine, Houston, TX 77030 USA

Correction to: *Nature Communications* 10.1038/s41467-018-03121-2, published online: 08 March 2018.

The original version of this article contained an error in the email address of the corresponding author Jun Qin. The correct email is jqin1965@126.com. The error has been corrected in the HTML and PDF versions of the article.

