# Supplementary material for: A proteomic landscape of diffuse-type gastric cancer
Source: Nat Commun. 2018 Mar 8;9:1012. doi: 10.1038/s41467-018-03121-2 (PMC5843664; doi:10.1038/s41467-018-03121-2)
Supplement: Supplementary file 1 — Supplementary Information [file 41467_2018_3121_MOESM1_ESM.pdf]

A Proteomic Landscape of Diffuse-type Gastric Cancer

Sai et al.

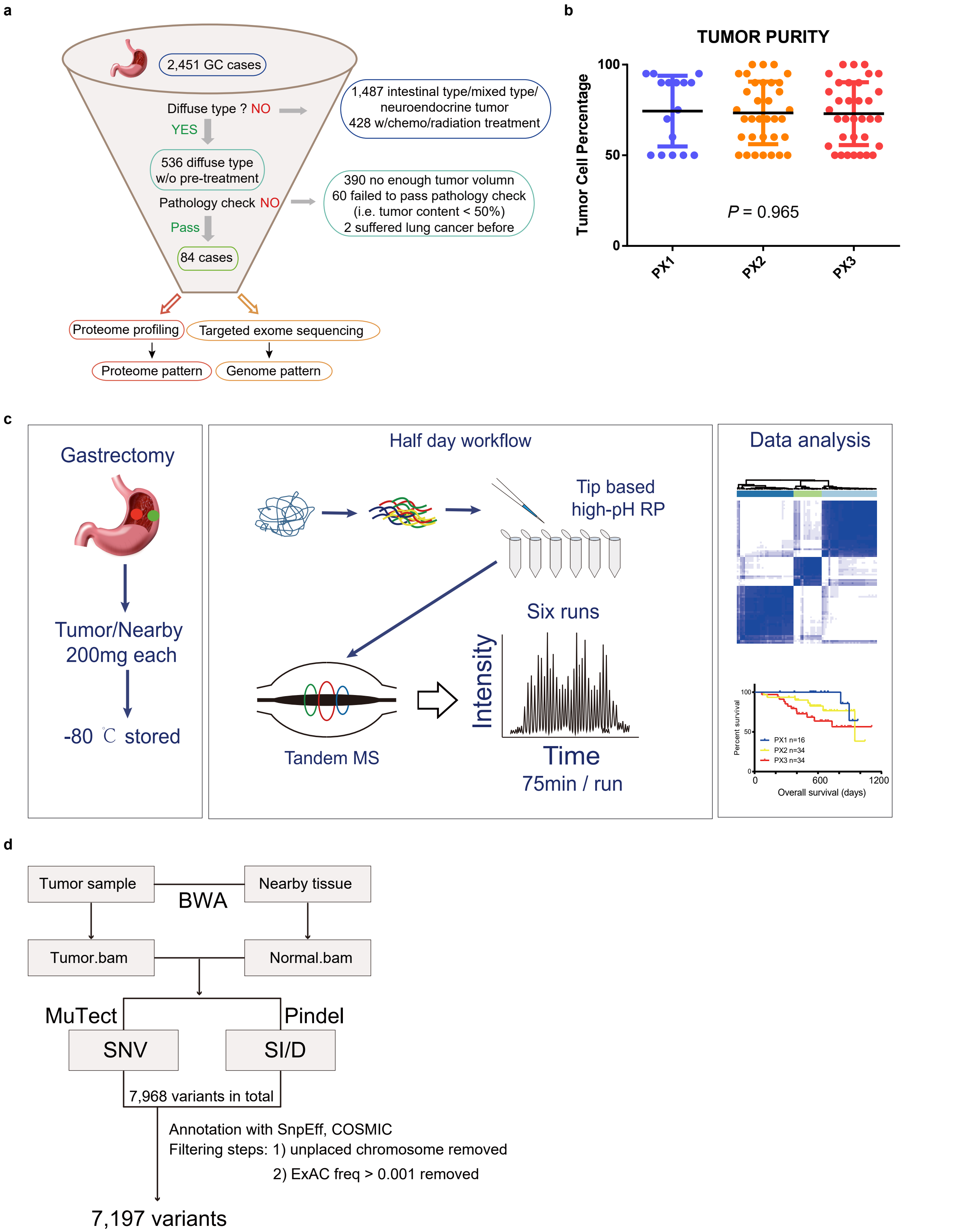

Supplementary Figure 1. Workflows for tumor sample procurements and proteomic analysis.
(a) Remark diagram detailing sample collection and quality control. The number of samples selected under each QC criteria is as indicated. 84 cases were included for the final analysis. (b) Tumor purity among three proteome subtypes showed no significant difference (One-way ANOVA, *P*=0.965). (c) Proteome analysis workflow. Tumors and paired nearby tissues were stored in the tissue bank at -80 degree till use. Protein was extracted and digested using trypsin. The tryptic digests were fractionated using the Tip based high-pH reverse-phase liquid chromatography into 9 fractions and then pooled into 6 MS runs to yield a half-day analysis workflow. (d) Variant calling pipelines of tumor and nearby tissues. Burrows-Wheeler Aligner (BWA) was used to map the reads to the reference genome hg19, MuTect and Pindel were used to call SNVs and small insertions and deletions (INDELs). In addition, variants were filtered against the ExAC database using a cutoff of 0.1%. SNVs and Indels were annotated using SnpEff based on UCSC known genes.

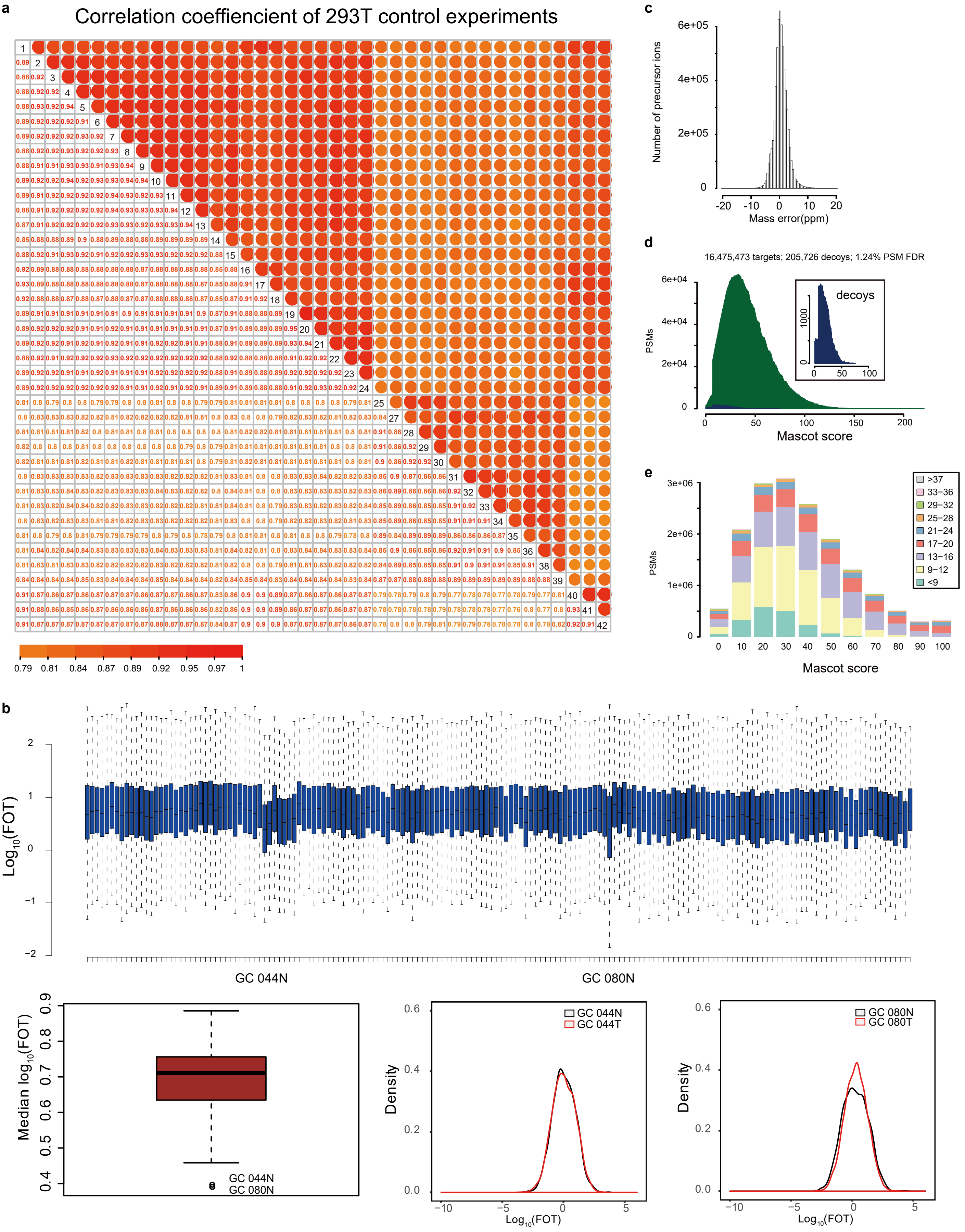

Supplementary Figure 2. MS platform quality control and global protein identifications.
(a) Pairwise Spearman's rank correlation coefficients for 42 MS runs of the tryptic digest of a 293T whole cell lysate every two days during the duration of the CNHPP project. (b) Distribution of $\log_{10}$ transformed FOT of the identified proteins in 168 samples (G4). Boxes range from lower quartile to upper quartile, and whiskers range from lower quartile-1.5 IQR (interquartile range) to upper quartile+1.5 IQR. Two samples (GC044N and GC080N) were lower than the lower quartile-1.5 IQR. Tumor/nearby tissue proteome ratio distributions of the two outliners show no bimodal distribution or exhibited significant skew (tailing). (c) Mass error distribution of the whole DGC dataset. (d) Global distribution of mascot ion scores for target and decoy PSMs at 1% PSM FDR. (e) Stacked bar plot of the peptide length distribution for global target PSMs.

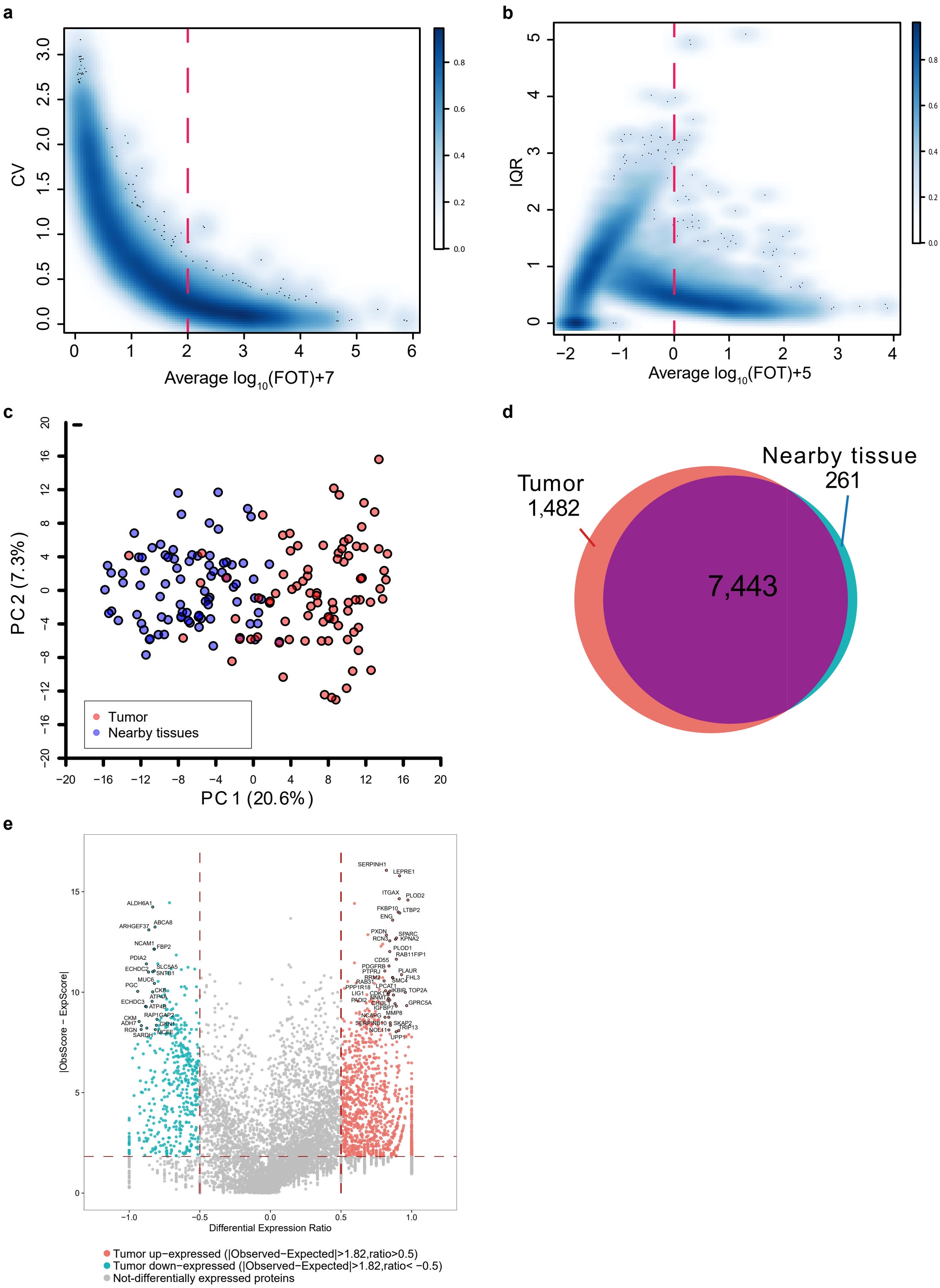

Supplementary Figure 3. Proteome quantification and the general proteome differences between tumor and the nearby tissue.

(a) Relationship between coefficient of variance (CV) and $\log_{10}$(FOT). When FOT reached more than 10^{-5}, CV significantly dropped with an average of 28%.(b) Relationship between interquartile range (IQR) and $\log_{10}$(FOT). Dramatic increase of IQR discontinued after FOT reached 10^{-5}. (c) Principal component analysis (PCA) using 3619 proteins to visualize tumor tissues and nearby tissues. Variances of PC1 and PC2 were 20.6% and 7.3%, respectively. (d) Detected protein numbers of tumor and the nearby tissue. (e) A volcano plot for displaying differentially expressed genes that are statistically significant between tumors and nearby tissues. The differential expression ratio (x axis) and the absolute value of observed score minus expected score (y axis) were plotted for each gene. Proteins with differential expression ratio more than 0.5 or less than -0.5 and |observed score – expected score|>1.82 (which equals FDR q value<0.01 by SAM) were significantly up/down-expressed proteins.

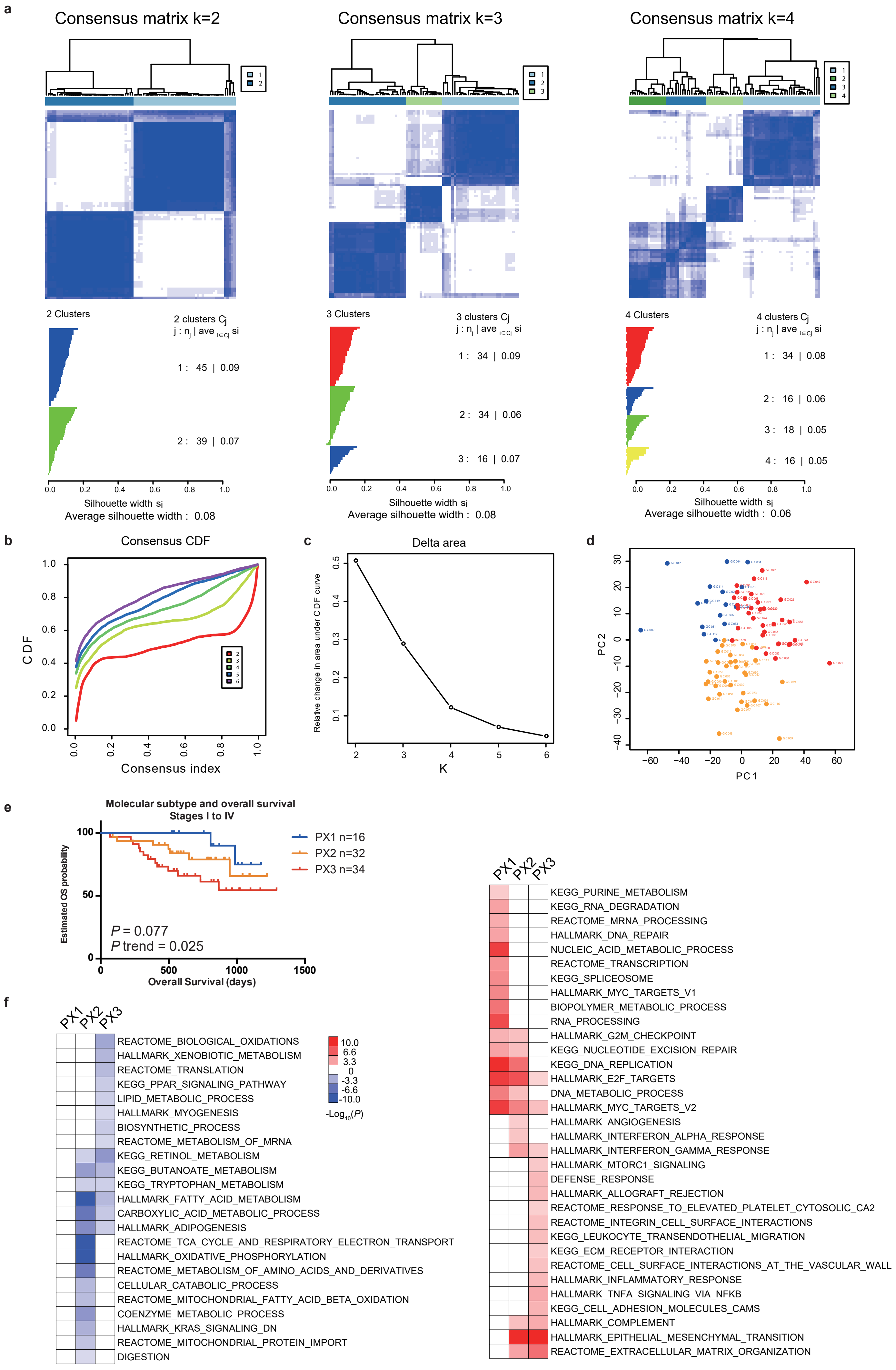
a
Consensus matrix k=2
Consensus matrix k=3
Consensus matrix k=4
2 Clusters
2 clusters Cj
j : nj | ave i∈Cj si
1 : 45 | 0.09
2 : 39 | 0.07
Silhouette width si
Average silhouette width : 0.08
3 Clusters
3 clusters Cj
j : nj | ave i∈Cj si
1 : 34 | 0.09
2 : 34 | 0.06
3 : 16 | 0.07
Silhouette width si
Average silhouette width : 0.08
4 Clusters
4 clusters Cj
j : nj | ave i∈Cj si
1 : 34 | 0.08
2 : 16 | 0.06
3 : 18 | 0.05
4 : 16 | 0.05
Silhouette width si
Average silhouette width : 0.06
b
Consensus CDF
CDF
Consensus index
c
Delta area
Relative change in area under CDF curve
K
d
PC2
PC1
e
Molecular subtype and overall survival
Stages I to IV
Estimated OS probability
Overall Survival (days)
PX1 n=16
PX2 n=32
PX3 n=34
P = 0.077
P trend = 0.025
f
PX1 PX2 PX3
REACTOME_BIOLOGICAL_OXIDATIONS
HALLMARK_XENOBIOTIC_METABOLISM
REACTOME_TRANSLATION
KEGG_PPAR_SIGNALING_PATHWAY
LIPID_METABOLIC_PROCESS
HALLMARK_MYOGENESIS
BIOSYNTHETIC_PROCESS
REACTOME_METABOLISM_OF_MRNA
KEGG_RETINOL_METABOLISM
KEGG_BUTANOATE_METABOLISM
KEGG_TRYPTOPHAN_METABOLISM
HALLMARK_FATTY_ACID_METABOLISM
CARBOXYLIC_ACID_METABOLIC_PROCESS
HALLMARK_ADIPOGENESIS
REACTOME_TCA_CYCLE_AND_RESPIRATORY_ELECTRON_TRANSPORT
HALLMARK_OXIDATIVE_PHOSPHORYLATION
REACTOME_METABOLISM_OF_AMINO_ACIDS_AND_DERIVATIVES
CELLULAR_CATABOLIC_PROCESS
REACTOME_MITOCHONDRIAL_FATTY_ACID_BETA_OXIDATION
COENZYME_METABOLIC_PROCESS
HALLMARK_KRAS_SIGNALING_DN
REACTOME_MITOCHONDRIAL_PROTEIN_IMPORT
DIGESTION
10.0
6.6
3.3
0
-3.3
-6.6
-10.0
-Log10(P)
PX1 PX2 PX3
KEGG_PURINE_METABOLISM
KEGG_RNA_DEGRADATION
REACTOME_MRNA_PROCESSING
HALLMARK_DNA_REPAIR
NUCLEIC_ACID_METABOLIC_PROCESS
REACTOME_TRANSCRIPTION
KEGG_SPLICEOSOME
HALLMARK_MYC_TARGETS_V1
BIOPOLYMER_METABOLIC_PROCESS
RNA_PROCESSING
HALLMARK_G2M_CHECKPOINT
KEGG_NUCLEOTIDE_EXCISION_REPAIR
KEGG_DNA_REPLICATION
HALLMARK_E2F_TARGETS
DNA_METABOLIC_PROCESS
HALLMARK_MYC_TARGETS_V2
HALLMARK_ANGIOGENESIS
HALLMARK_INTERFERON_ALPHA_RESPONSE
HALLMARK_INTERFERON_GAMMA_RESPONSE
HALLMARK_MTORC1_SIGNALING
DEFENSE_RESPONSE
HALLMARK_ALLOGRAFT_REJECTION
REACTOME_RESPONSE_TO_ELEVATED_PLATELET_CYTOSOLIC_CA2
REACTOME_INTEGRIN_CELL_SURFACE_INTERACTIONS
KEGG_LEUKOCYTE_TRANSENDOTHELIAL_MIGRATION
KEGG_ECM_RECEPTOR_INTERACTION
REACTOME_CELL_SURFACE_INTERACTIONS_AT_THE_VASCULAR_WALL
HALLMARK_INFLAMMATORY_RESPONSE
HALLMARK_TNFA_SIGNALING_VIA_NFKB
KEGG_CELL_ADHESION_MOLECULES_CAMS
HALLMARK_COMPLEMENT
HALLMARK_EPITHELIAL_MESENCHYMAL_TRANSITION
REACTOME_EXTRACELLULAR_MATRIX_ORGANIZATION

Supplementary Figure 4. Consensus clustering of DGC into 3 subtypes based on proteome patterns.
(a) The upper panel shows consensus matrices of the 84 GC samples from k=2 to k=4. Consensus clustering was performed on 2,538 proteins (D5). The lower panel shows the silhouette plots. (b) The cumulative distribution function (CDF) plots corresponding to the consensus matrices from k=2 to k=4. (c) Delta plot assessing changes in the consensus CDF area to search for ideal number of clusters. As k increases, the area under the CDF is hypothesized to increase markedly until k reaches the ideal value. Three was considered as the ideal value based on both visual inspection of the consensus matrix and the change of the area under the CDF was close to zero when k increased from 3 to 4. (d) Principal component analysis (PCA) using 2,538 proteins to separate PX1-3. (e) Association of molecular subtypes with overall survival in all 82 patients (Kaplan-Meier analysis, *P* value from Log-rank test).(f) Pathways significantly decreased (left) or elevated (right) in PX1-3 in tumors compared with nearby tissues.

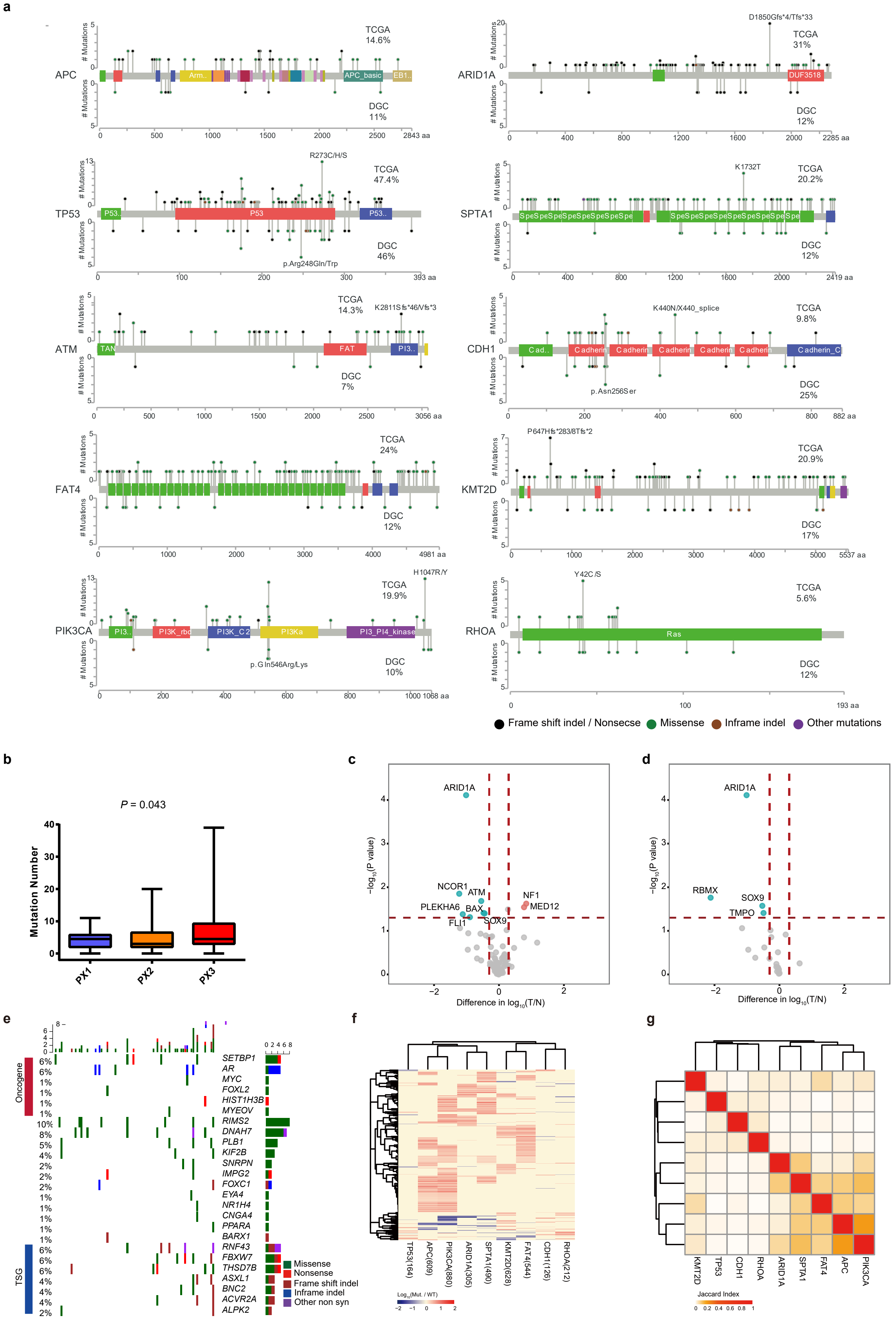
a
APC
TCGA 14.6%
DGC 11%
Arm..
APC_basic
EB1..
2843 aa
ARID1A
D1850Gfs*4/Tfs*33
TCGA 31%
DGC 12%
DUF3518
2285 aa
TP53
R273C/H/S
TCGA 47.4%
DGC 46%
P53..
P53
p.Arg248Gln/Trp
393 aa
SPTA1
K1732T
TCGA 20.2%
DGC 12%
2419 aa
ATM
TCGA 14.3%
K2811Sfs*46/Vfs*3
DGC 7%
TAN
FAT
PI3..
3056 aa
CDH1
K440N/X440_splice
TCGA 9.8%
DGC 25%
Cad..
Cadherin
Cadherin_C
p.Asn256Ser
882 aa
FAT4
TCGA 24%
DGC 12%
4981 aa
KMT2D
P647Hfs*283/8Tfs*2
TCGA 20.9%
DGC 17%
5537 aa
PIK3CA
H1047R/Y
TCGA 19.9%
DGC 10%
PI3K_rbd
PI3K_C2
PI3Ka
PI3_PI4_kinase
p.Gln546Arg/Lys
1000 1068 aa
RHOA
Y42C/S
TCGA 5.6%
DGC 12%
Ras
193 aa
Mutations
Frame shift indel / Nonsecse
Missense
Inframe indel
Other mutations
b
P = 0.043
Mutation Number
PX1
PX2
PX3
c
ARID1A
NCOR1
ATM
PLEKHA6
BAX
FLI1
SOX9
NF1
MED12
−log10(P value)
Difference in log10(T/N)
d
ARID1A
RBMX
SOX9
TMPO
−log10(P value)
Difference in log10(T/N)
e
Oncogene
TSG
SETBP1
AR
MYC
FOXL2
HIST1H3B
MYEOV
RIMS2
DNAH7
PLB1
KIF2B
SNRPN
IMPG2
FOXC1
EYA4
NR1H4
CNGA4
PPARA
BARX1
RNF43
FBXW7
THSD7B
ASXL1
BNC2
ACVR2A
ALPK2
Missense
Nonsense
Frame shift indel
Inframe indel
Other non syn
f
TP53(164)
APC(609)
PIK3CA(880)
ARID1A(305)
SPTA1(490)
KMT2D(628)
FAT4(544)
CDH1(126)
RHOA(212)
Log10(Mut. / WT)
g
KMT2D
TP53
CDH1
RHOA
ARID1A
SPTA1
FAT4
APC
PIK3CA
Jaccard Index

Supplementary Figure 5. Exome sequencing of hotspot genes in DGC.

(a) Lollipop plots showing the type and location of mutations in 10 representative proteins, for example, *TP53, ATM, ARID1A* and *etc*. (b) Gene mutation numbers of PX1, PX2 and PX3 (One-way ANOVA, *P*=0.043), the box plots show the median, 25th and 75th percentile values (horizontal bar, bottom and top bounds of the box), and the highest and lowest values (top and bottom whiskers, respectively). (c) A volcano plot to illustrate significantly altered protein expressions for genes with nonsynonymous mutations compared with the wild type. Up-regulated and down-regulated genes are indicated in red and blue, respectively. *P* values were calculated using Wilcoxon rank sum test. (d) A volcano plot to illustrate significantly altered protein expressions for genes with protein-truncating mutations compared with the others. Up-regulated and down-regulated genes are indicated in red and blue, respectively. *P* values were calculated using Wilcoxon rank sum test. (e) OncoPrint of mutated genes whose protein expressions were undetectable. Bars on top and to the right of the graph showed the number of non-synonymous mutations in each patients and genes, respectively. (f) Hierarchical clustering of altered proteomes associated with nine high-frequent mutations compared with wild types. Colors indicate ratio of median tumor expression in mutated samples to that in the wild type samples, numbers in brackets at the bottom indicate the number of altered proteins associated with each gene mutation.(g) A heat map of altered proteome similarity for the nine high-frequency mutations. Similarity between each pairs of mutations was estimated with the pair-wise Jaccard index.

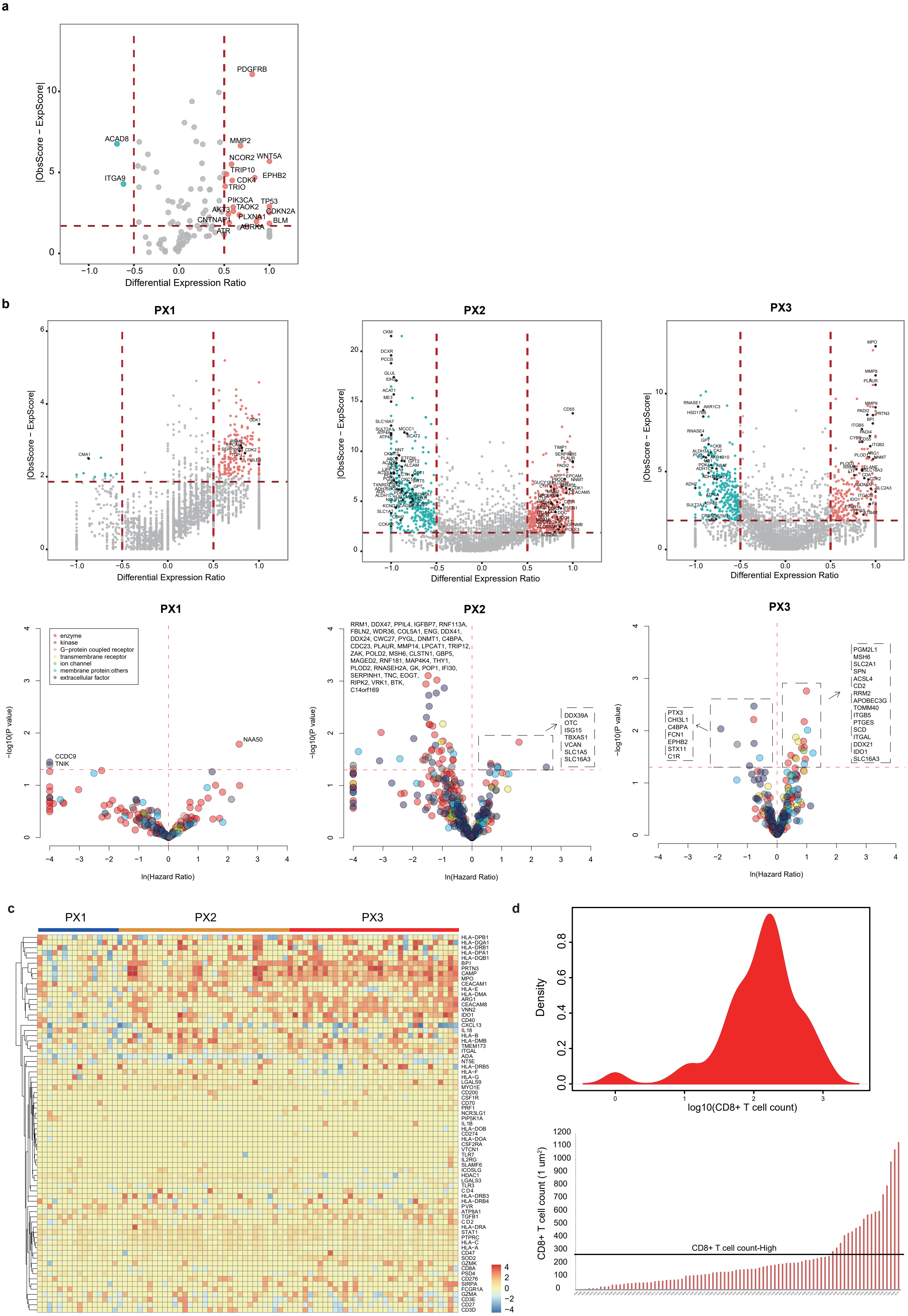

a
|ObsScore – ExpScore|
Differential Expression Ratio
PDGFRB
ACAD8
MMP2
NCOR2
WNT5A
ITGA9
TRIP10
EPHB2
CDK4
TRIO
PIK3CA
TP53
AKT3
TAOK2
CDKN2A
CNTNAP1
PLXNA1
BLM
AURKA
ATR
b
PX1
PX2
PX3
Differential Expression Ratio
|ObsScore – ExpScore|
−log10(P value)
ln(Hazard Ratio)
enzyme
kinase
G-protein coupled receptor
transmembrane receptor
ion channel
membrane protein:others
extracellular factor
CCDC9
TNIK
NAA50
RRM1, DDX47, PPIL4, IGFBP7, RNF113A, FBLN2, WDR36, COL5A1, ENG, DDX41, DDX24, CWC27, PYGL, DNMT1, C4BPA, CDC23, PLAUR, MMP14, LPCAT1, TRIP12, ZAK, POLD2, MSH6, CLSTN1, GBP5, MAGED2, RNF181, MAP4K4, THY1, PLOD2, RNASEH2A, GK, POP1, IFI30, SERPINH1, TNC, EOGT, RIPK2, VRK1, BTK, C14orf169
DDX39A
OTC
ISG15
TBXAS1
VCAN
SLC1A5
SLC16A3
PTX3
CHI3L1
C4BPA
FCN1
EPHB2
STX11
C1R
PGM2L1
MSH6
SLC2A1
SPN
ACSL4
CD2
RRM2
APOBEC3G
TOMM40
ITGB5
PTGES
SCD
ITGAL
DDX21
IDO1
SLC16A3
c
PX1
PX2
PX3
d
Density
log10(CD8+ T cell count)
CD8+ T cell count (1 um²)
CD8+ T cell count-High

Supplementary Figure 6. Nominating potential druggable protein candidates and representative immune-related protein expressions in PX1-3.

(a) A volcano plot was used for displaying difference between tumor and nearby tissues of genomic discovered druggable candidates. Proteins with |differential expression ratio| >0.5 and |observed score - expected score|>1.82 (which equals FDR q value<0.01 by SAM) were defined as significantly up/down-expressed proteins.(b) Differential expressed druggable protein candidates in PX1-3, respectively (upper three plots) and their associations with overall survival (bottom three plots). The Ln (Hazard ratio) (x-axis) and $\log_{10}$ (*P* value) (y-axis) were calculated from Cox proportional hazards regression analysis. Large dots depict proteins overexpressed in tumors, small dots depict proteins that are not overexpressed. (c) A heat map for displaying proteins listed for immunotherapy in REFs (Melero et al., 2015; Smyth et al., 2016) and (Pardoll, 2012). Values of Log_{10} (T/N) are plotted, red means over-expressed and blue means down-expressed. (d) CD8 positive cell count result. Peak of the distribution of CD8+ cell count number is 171.4 (upper plot), and we chose the first significant change (at least 10% higher than the former number) after the peak as the threshold for CD8-high samples, which is 298 per μm^2.

Supplementary Table. The three clusters and patient characteristics

Variable	PX1	PX2	PX3	*P* value
Patient Number	16 (19.0%)	34 (40.5%)	34 (40.5%)	
Gender [a]				
Male	11 (68.8%)	17 (50.0%)	25 (71.4%)	0.116
Female	5 (31.3%)	17 (50.0%)	9 (26.5%)	
Median Age [b]	62 (50-78)	55 (26-84)	60 (25-87)	0.166
Tumor Site				
Cardia, gastroesophageal junction	6 (37.5%)	12 (35.3%)	4 (11.8%)	**<0.001**
Body	7 (43.8%)	17 (50.0%)	9 (26.5%)	
Antrum	3 (18.8%)	5 (14.7%)	21 (61.8%)	
Signet Ring Cell				
Negative	12 (75.0%)	15 (44.1%)	21 (61.8%)	0.105
Positive	4 (25.0%)	19 (55.9%)	13 (38.2%)	
Nearby normal tissue				
Superficial gastritis	6 (37.5%)	27 (79.4%)	10 (29.4%)	**<0.001**
Atrophic gastritis	5 (31.3%)	5 (14.7%)	11 (32.4%)	
Intestinal metaplasia, dysplasia	4 (25.0%)	2 (5.9%)	12 (35.3%)	
Not detected	1 (6.3%)	0 (0%)	1 (2.9%)	
AJCC stage 7th				
Ib	0 (0%)	1 (2.9%)	4 (11.8%)	0.255
II	5 (31.3%)	10 (29.4%)	5 (14.7%)	
III	10 (62.5%)	22 (64.7%)	25 (73.5%)	
IV	1 (6.3%)	1 (2.9%)	0 (0.0%)	
EBV				
Positive	0 (0%)	2 (5.9%)	4 (11.8%)	0.555
Negative	16 (100%)	32 (94.1%)	30 (88.2%)	
Adjuvant chemotherapy				
With [c]	15 (93.8%)	26 (76.5%)	25 (67.6%)	0.284
Without	1 (6.3%)	8 (23.5%)	9 (32.4%)	
Median Mutation Number [b]	4 (0-11)	3 (0-20)	5 (0-39)	**0.043**
MSI Status				
Microsatellite stable	16 (100%)	34 (100%)	29 (85.3%)	**0.022**
Microsatellite instable	0 (0%)	0 (0%)	5 (14.7%)	

[a] Chi-square was used, [b] One-way ANOVA test was used, for others all used Fisher's exact test.
[c] Patients proceed at least one cycle of adjuvant chemotherapy. Significant data are emphasized in bold.
