# Supplementary material for: A proteomic landscape of diffuse-type gastric cancer
Source: Nat Commun. 2018 Mar 8;9:1012. doi: 10.1038/s41467-018-03121-2 (PMC5843664; doi:10.1038/s41467-018-03121-2)
Supplement: Supplementary file 3 — Description of Additional Supplementary Files [file 41467_2018_3121_MOESM3_ESM.pdf]

Description of Additional Supplementary Files

File Name: Supplementary Data 1
Description: Clinical features, histopathology annotation and follow-up information of all 84 patients.

File Name: Supplementary Data 2
Description: Proteome datasets, filter criteria and targeted sequencing result.

File Name: Supplementary Data 3
Description: Differentially expressed proteins in whole cohort and each clusters.

File Name: Supplementary Data 4
Description: GSEA pathway enrichment among three clusters.

File Name: Supplementary Data 5
Description: Pathway enrichment of mutations among three clusters and mutation-protein correlation analysis.

File Name: Supplementary Data 6
Description: Known druggable and prognosis unfavorable targets analysis.
